# Effect of light intensity in the nest site on eggshell pigmentation in a hole-nesting passerine

**DOI:** 10.1038/s41598-023-36658-4

**Published:** 2023-06-16

**Authors:** Katarzyna Malinowska, Klaudia Szala, Paweł Podkowa, Adrian Surmacki

**Affiliations:** 1 Population Ecology Lab, Faculty of Biology, Adam Mickiewicz University, Uniwersytetu Poznańskiego 6, 61-614 Poznań, Poland; 2 Department of Avian Biology and Ecology, Faculty of Biology, Adam Mickiewicz University, Uniwersytetu Poznańskiego 6, 61-614 Poznań, Poland; 3 Institute of Environmental Sciences, Jagiellonian University, Gronostajowa 7, 30-387 Kraków, Poland

**Keywords:** Behavioural ecology, Evolutionary ecology, Zoology

## Abstract

Daylight is an important factor necessary for the proper embryonic development of birds, which raises the question, what happens when birds nest in relatively dim sites? The study experimentally tested whether there is a relationship between light conditions at the nesting site and the protoporphyrin-based pigmentation in the eggshell of the Great Tit (*Parus major*). We hypothesised that at lower light levels, eggs are less pigmented to increase the amount of light reaching the embryo. Our study system consisted of two types of nest boxes: "dark", in which the only source of light was the entrance hole, and "bright", which had two additional side windows. Photographs of clutches taken during the incubation period were used to quantify eggshell pigmentation. Multispectral image analyses were performed to measure variables correlating with protoporphyrin content, such as spot brightness, average spot size, spotting coverage, and spot red chroma. Repeatability analysis indicated that eggshell colouration characteristics were significantly and moderately repeatable between eggs from a single clutch, which suggests that they are under genetic and environmental control. However, none of the pigmentation traits differed significantly between the two types of nest boxes. We speculate about other ecological aspects that might have influenced the observed variability in eggshell pigmentation.

## Introduction

Eggshells of many bird species exhibit a great variety of colours and patterns, which are effects of pigment deposition^1^. In most cases, eggs are pigmented by biliverdin, which produces a blue-green hue, and/or protoporphyrin, which gives a rusty-brown colour^1^, but there are also a few rare pigments^2,3^. Over the last decades, several not mutually exclusive hypotheses have been developed to explain the evolution of variation in eggshell colouration. These include eggshell strengthening^4^, camouflage^5,6^, recognising own clutch and protection against brood parasitism^7,8^, thermoregulation^9,10^, and sexual selection^11^, to mention just the most common hypotheses.

An interesting study^12^ drew attention to the fact that the eggshell transmits natural light, which eventually reaches the embryo. Moreover, there are many premises that light-filtering eggshells may affect developing birds in many ways^12^. In many cases, the effect of light can be regarded as positive. For example, it accelerates embryo development, provides energy for DNA repair processes (photo-reactivation), promotes functional lateralisation of cerebral hemispheres, and induces the antibacterial function of eggshell pigments on the egg surface^12^. On the other hand, UV-B radiation (290–320 nm) is a harmful factor because it damages the DNA of the embryo^12^. Thus, eggshell pigmentation and thickness form a filter modulating the amount and spectrum of light that reaches the embryo inside the egg^13^. The transmission of light through the calcium matrix increases linearly with light wavelength, while pigments act as filters blocking some wavebands and letting others through^13^. In general, the overall light transmittance decreases with increasing pigmentation and eggshell thickness^13^.

Bird nest sites are characterised by a wide variety of light conditions, which results from nest construction (domed and cavity nests vs. open cups), as well as from vegetation structures^13^. Therefore, depending on the nest light conditions, various processes can shape the evolution of the appearance of eggshells in different species. Open nesters need to limit the amount of harmful UV-B light reaching the embryo and avoid overheating^14,15^, whereas dim light conditions inside cavities should promote higher transmission of light through eggshells, to trigger the beneficial effect of light on the embryo. In addition, brighter colours could increase the contrast between eggs and nest background, and thus visibility to the parents^13^. These predictions have been supported by the results of comparative analyses. Cavity-nesting species generally tend to lay less pigmented or unpigmented eggs, while open nesters’ eggs are often variously patterned^14,16–18^.

In this study, we have made the first attempt to test whether light conditions in the nest site influence eggshell colouration at the population level in a hole-nesting passerine. We used an experimental setup with nest boxes, in which we controlled the amount of entering sunlight. Boxes were occupied by a secondary hole-nesting species, the Great Tit (*Parus major*), which lays eggs with a light-coloured eggshell background variously spotted with protoporphyrin^19^. Birds could choose to nest in standard (“dark”) or artificially brightened (“bright”) boxes. Our main aim was to test experimentally whether eggshell pigmentation depends on light intensity inside the nest box, while controlling for several ecological traits that could potentially affect eggshell colouration. In particular, we estimated the effect of eggshell thickness, the first egg laying date, and the clutch size on the variation of eggshell patterning. We expected that females breeding in dark nest boxes would have less pigmented eggshells, to compensate for the light deficiency in dimmer conditions**.** Moreover, we calculated the intra-clutch repeatability of eggshell patterning, to assess to what extent the pattern is consistent between eggs laid by the same female.

## Results

### Effect of nest box type on eggshell pigmentation

Generalised Additive Mixed Models (GAMMs) revealed that none of the eggshell colouration characteristics differed significantly between dark and bright nest boxes: *percent spots* (*z* = 1.66, *p* = 0.10), *spot brightness* (*z* =  − 0.06, *p* = 0.95), *average spot size* (*t* = 0.69, *p* = 0.49) and *spot Rchroma* (*z* = -0.46, *p* = 0.64) (Table 1).

**Table 1:** Summary table for Generalized Additive Mixed Models (GAMMs) fitted to Great Tit eggshell pigmentation traits and accounted for the nest box type (standard “dark” and artificially brightened “bright” nest boxes), clutch size, date of the first egg and eggshell thickness.

Parametric coefficients																
Term	Percent spots				Spot Rchroma				Average spot size				Spot brightness			
	Estimate	SE	*z*	*P*	Estimate	SE	*z*	*p*	Estimate	SE	*t*	*P*	Estimate	SE	*z*	*p*
(Intercept)	−1.69	0.24	−7.12	**< 0.01**	−0.54	0.07	−8.06	**< 0.01**	2.96	0.64	4.64	**< 0.01**	−1.05	0.56	−1.87	0.06
Clutch size	0.04	0.02	2.02	**0.04**	0.00	0.01	−0.41	0.68	0.05	0.03	1.97	**0.05**	−0.03	0.03	−0.85	0.40
Nest box type	0.12	0.07	1.66	0.10	−0.01	0.02	−0.46	0.64	0.06	0.09	0.69	0.49	0.01	0.11	0.06	0.95
Thickness									239.92	88.38	2.72	**0.007**	233.40	68.46	3.41	**< 0.001**

Approximate significance of smooth terms																
	edf	Ref.df	Chi-sq	*p*	edf	Ref.df	Chi-sq	*p*	edf	Ref.df	*F*	*p*	edf	Ref.df	Chi-sq	*p*
s(First egg date)	1	1	3.22	0.07	4.38	4.43	17.64	**0.002**	1.00	1.00	0.98	0.32	1.00	1.00	0.04	0.85
s(Nest box ID)	25.4	28.00	280.22	**< 0.01**	23.46	28.00	493.01	**< 0.01**	24.63	28.00	7.12	**< 0.01**	27.03	28.00	806.12	**< 0.01**
s(Thickness)	2.40	3.10	4.67	0.22	1.62	2.05	0.92	0.61								
	R^2^_adj_ = 0.536				R^2^_adj_ = 0.789				R^2^_adj_ = 0.442				R^2^_adj_ = 0.726			

Significant values are in bold.

**Figure 1:**
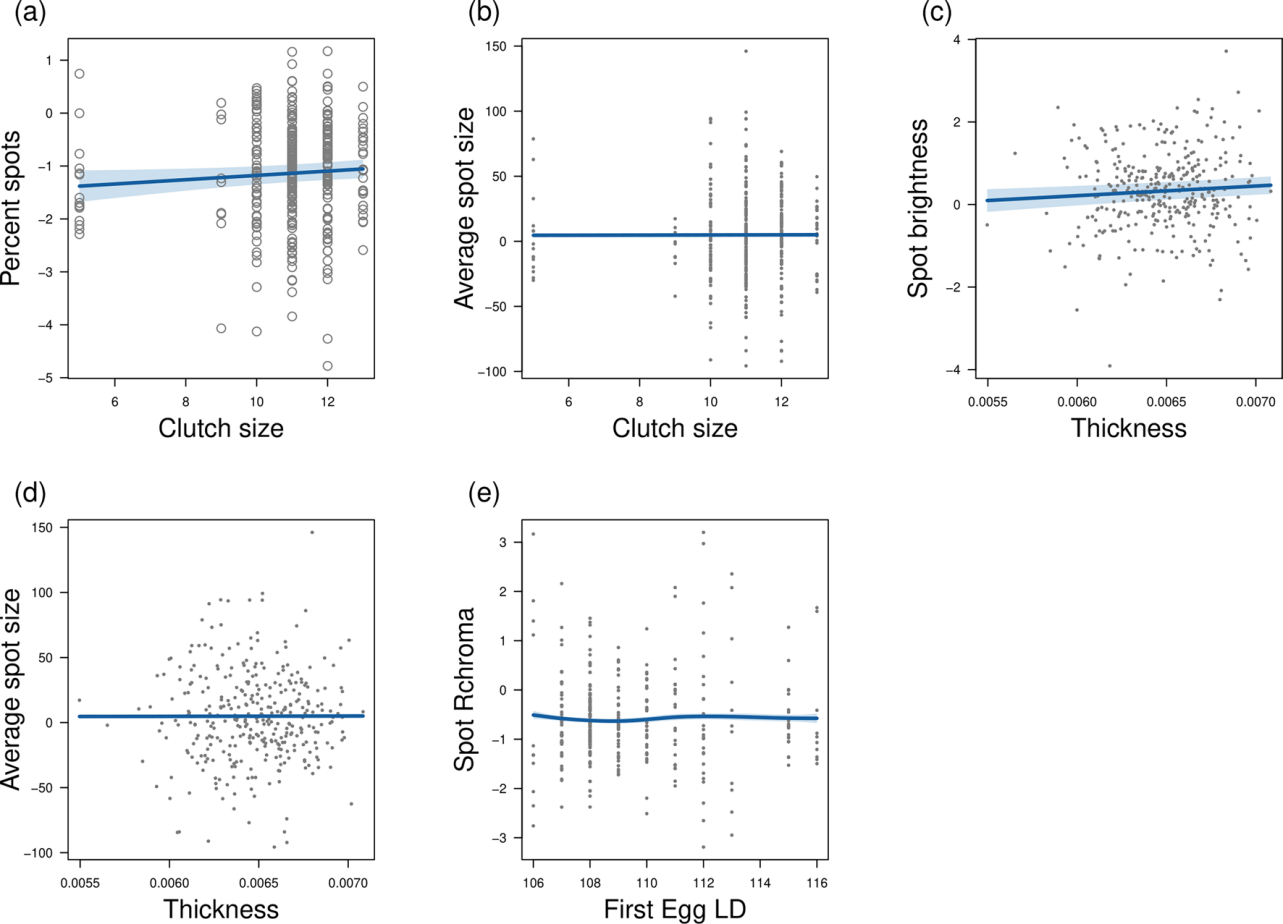
(**a**–**e**) Conditional plots visualising significant effects of Great Tit egg characteristics on pigmentation traits presented on the scale of the linear predictor. Shaded areas indicate 95% confidence intervals and points represent partial residuals. LD = laying date.

However, data showed (Table 1 and Supplementary Fig. S1) that clutch size has a positive effect on *percent spots* (*z* = 2.02, *p* = 0.04) (Fig. 1a) and a marginally significant effect on *average spot size* (*t* = 1.97, *p* = 0.05) (Fig. 1b). We also found a positive association between eggshell thickness and both *spot brightness* (*z* = 3.41, *p* < 0.001) (Fig. 1c) and *average spot size* (*t* = 2.72, *p* = 0.007) (Fig. 1d). Besides, data revealed a nonlinear relationship (chi-square = 17.64, *p* = 0.002) between the laying date of the first egg and *spot Rchroma* (Fig. 1e).

### Repeatability of traits

All studied egg traits were significantly repeatable within clutches (Table 2), but egg dimensions were on average more repeatable than colouration traits (0.778 vs. 0.638). According to the classification of repeatability values^20^, the repeatability of all egg dimensions was good (*R* ≥ 0.75). Among eggshell pigmentation traits, *spot brightness* and *percent spots* were moderately repeatable (0.75 > *R* > 0.50), *spot Rchroma* was the most repeatable of all the traits studied (*R* = 0.852), whereas *average spot size* had poor repeatability (*R* < 0.50).

**Table 2:** Results of the repeatability analysis of Great Tit egg dimensions and pigmentation traits.

Trait	Repeatability	Upper 95% CI	Lower 95% CI	*p*
Egg volume	0.818	0.707	0.886	< 0.001
Egg length	0.769	0.646	0.844	< 0.001
Egg width	0.747	0.616	0.834	< 0.001
Spot Rchroma	0.794	0.680	0.859	< 0.001
Spot brightness	0.738	0.609	0.818	< 0.001
Percent spots	0.564	0.415	0.676	< 0.001
Average spot size	0.456	0.298	0.583	< 0.001

## Discussion

Contrary to our predictions, we found no significant effect of light conditions in the nest site on variation in eggshell pigmentation. We are confident that the obtained results are not artefacts due to the flawed methodology of eggshell colouration measurements. Standardised digital photography is an accurate and objective method widely used to score eggshell pigmentation^19,21^. Digital photography has an advantage over other methods because it is not based on subjective patterning scores^22^ and, contrary to spectrophotometry, it is capable of embracing the entire egg surface, including eggs with complicated patterning^23^. Moreover, our previous study showed that the light intensity manipulation used in this study was efficient enough to create distinctly different light conditions. The light intensity in the “bright” boxes is about 50 times higher than in the “dark” ones^24^.

The results of our study did not indicate any linkage between nest site light conditions and eggshell pigmentation, regardless of potential mechanisms of such a relationship. All measures of eggshell colouration were significantly intra-female repeatable, and the repeatability of spot red chroma could be even classified as high^20^. Our study confirms results from earlier studies on the Great Tit^25^ and other species with egg pigmentation based on protoporphyrin^26^, which indicated that the repeatability of eggshell colouration traits ranges approximately from 0.5 to 0.9. Since repeatability reflects the upper limit of heritability of phenotypic variation^27^, we can conclude that eggshell colouration in the studied Great Tit population is under both environmental and genetic control^28^. Another study concerning this issue was carried out on the Great Tit population in Wytham Woods, which showed that eggshell patterning traits present a certain level of heritability and are rather weakly influenced by environmental conditions^28^. However, a direct comparison with our study is not possible, as the variation of eggshell patterning was assessed using different methods. Moreover, in the present study, we applied within-clutch repeatability, whereas in Wytham Woods between-clutch repeatability was used. Furthermore, these two populations live in distant geographical locations that may differ in the mechanisms responsible for eggshell patterning^28^.

The high variability of eggshell colouration in some bird species is supposed to evolve as an adaptation to particular environmental conditions. A classic example is the Common Cuckoo (*Cuculus canorus*), with several genetic lineages of females specialised to produce eggs coloured to match eggs of their preferred hosts^29^. A similar mechanism exists even in species with more subtle variation in eggshell colouration. A recent study has shown that females inherit a preference for particular nest habitat, which provides the best camouflage for a given eggshell pigmentation^30^. Assuming the great importance of light for embryo development, an analogous selection pressure on eggshell pigmentation in Great Tit with regard to light intensity in the nesting site could be expected.

Despite the obvious variability in eggshell colouration, we found no differences in colouration traits between dark and bright nest boxes. It is possible that the potential benefits of better light transmission are not significant enough to cause different levels of pigment deposition between dark and bright nest boxes or that other functions of pigmentation are more important in the case of the Great Tit. On the other hand, certain benefits of improved illumination have been observed. It is known that the population of Great Tits shows clear preferences for brighter nest boxes^24^. In addition, offspring raised in bright nest boxes are in a slightly better condition compared to dark boxes conspecifics, as they have higher immunocompetence and fledge earlier^31^. The lack of relationship between eggshell colouration and nest box type revealed by our experiment could also result from the sensibility of the studied species to light. It is probable that even small amounts of light passing through the eggshell may induce beneficial effects on the embryo. Although this hypothesis awaits future studies, such a possibility is likely since the cavity-nesters have evolved in a very constrained light regime^32^. Another important aspect is the behavioural response of Great Tits to nest box illumination. Individuals from the studied population preferred to nest in bright nest boxes, but birds from dark nest boxes actively compensated for the light deficiency by building significantly higher nests (i.e. closer to the entrance hole)^24^. Thus, the response to varied light levels could be rather behavioural than physiological.

Our study was the first attempt to experimentally investigate the relationships between solar radiation in the nest site and eggshell pigmentation in wild birds. However, there are some correlative studies that link egg colouration intensity with the protection of the embryo from UV-B radiation. The first one was devoted to populations of Village Weaver (*Ploceus cucullatus*), which builds a woven domed nest, whose walls partly transmit sunlight. It has been demonstrated that populations exposed to a higher intensity of solar UV radiation have more pigmented eggs, as compared to a population from area situated at higher latitudes and with more frequent cloud cover^33^. Similarly, in a ground-nesting species, Kentish Plover (*Charadrius alexandrinus*), clutches laid in areas of higher UV radiation were darker^34^. Also Reed Warbler (*Acrocephalus scirpaceus*) eggshells were brighter when the clutch was laid during heavy rains, and hence, presumably, under lower illumination due to cloud cover^35^. The above studies suggest that eggshell pigmentation has a protective function, which is important in species breeding in open areas or areas where a threat from UV radiation is high. It is important to keep in mind, however, that due to the correlative nature of these studies, other factors could interfere with eggshell pigmentation, aside from light, e.g. geographical location, ambient temperature, precipitation or food availability.

The observed variation in eggshell pigmentation could also reflect female body condition, which was not controlled in our study. One study reported^36^ that lighter Great Tit females lay more pigmented eggs, but this relationship was not confirmed in other studies concerning e.g. body size^25,37,38^. Some studies indicate that females that lay darker eggs may be less healthy and suffer from physiological stress, because of the oxidative properties of protoporphyrin^38,39^, and this could in turn have a negative impact on the quality of eggs and chicks^40^. Another factor that could potentially affect eggshell pigmentation is female age. In the Blue Tit (*Cyanistes caeruleus*), a closely related species, older females tend to lay brighter eggs^39^.

Our study revealed that eggs from larger clutches tended to have bigger spots, which covered a slightly higher percentage of the eggshell surface. Previous research provided mixed results on this relationship. For example, an opposite relationship was shown^38^: bigger clutches had lower concentrations of protoporphyrin, whereas other study^36^ did not find any significant relationship. A possible explanation of the pattern found in the present study is that females laying bigger clutches are more deficient in calcium and use the pigment as a structural reinforcement of the eggshell^4^. This hypothesis, however, assumes that more protoporphyrin should be built into the eggs with thinner shells^4,41^. We found that the thickness and eggshell pigmentation traits are correlated: eggs with bigger and brighter spots had significantly thicker eggshells. Thus, our results partly support and partly contradict the eggshell reinforcement hypothesis. One possible interpretation is that females in superior conditions can afford to produce eggshells that are both thick and intensively pigmented. It is also possible that in eggs with thicker eggshells, the pigment gets into the deeper shell layers, and this could result in brighter spots but still keep its efficient filtering function. Alternatively, the trade-off between the number of spots and their colouration is possible. Assuming that protoporphyrin is a limited resource, females that produce more densely spotted eggs must reduce their colour intensity.

We found that the spot red chroma intensity varied nonlinearly with the first egg laying date, as it decreased from the beginning of the season to the 19th of April, next increased until the 22nd of April, and slightly decreased later (Supplementary Fig. S1). Similar studies on the same species have shown that egg pigmentation intensity and spotting coverage decrease during the season^28,42^, or did not find any relationship^36^. Moreover, Great Tits tend to produce more pigmented eggs in poorer environmental conditions: during colder weather, food shortage, and higher breeding density^42^. Although we did not measure these variables, the red chroma fluctuating pattern may be explained by changing environmental conditions during the season, especially varying temperature.

One of the limitations of our study is the fact that the experiment was conducted on a Great Tit population breeding in nest boxes, where light conditions may significantly differ from those experienced in natural hollows. Although this topic remains largely unexplored, it is presumed that natural hollows are generally very dark, at least at dawn^43,44^. On the other hand, they can be brighter, depending on the time of day and size, shape, orientation of the entrance, and whether there are additional openings such as cracks and crevices in the trunk^44^. Therefore, we decided to use two extreme light intensities to investigate its potential impact, although these may not accurately reflect the conditions Great Tits naturally experience in the nesting site. However, the use of artificial cavities has a number of advantages over natural hollows, which are scarce in most managed forests. They provide unobstructed and quick access to the nest and eggs, the possibility to monitor broods^45^, and to manipulate physical factors, e.g., light or temperature^46^. Another limitation of our study is that it was performed during only one season. Conducting the experiment in subsequent years under varied environmental conditions could provide a more comprehensive understanding of how eggshell pigmentation interacts with light conditions at the nesting site. Moreover, the design of our study was inadequate to check whether light intensity in the nesting site could shape eggshell pigmentation on a longer time scale, since we investigated only one generation of birds and lacked pedigree data. Our Great Tit population was exposed to extreme light conditions for a period of time that was too short (three years since the nest boxes were hung) to cause significant evolutionary changes. Additionally, studies on other cavity-nesting species may be beneficial, as light sensitivity may be species-specific.

In the present study, we found no relationship between light intensity at the nesting site and eggshell pigmentation traits in the Great Tit. Repeatability values of eggshell pigmentation characteristics suggest that they may depend on both genetic and environmental factors. Many other factors, such as the breeding female condition, which was not controlled in this study, could contribute to much of the observed variation in eggshell colouration. Moreover, our study did not find unequivocal support for the eggshell reinforcement function of protoporphyrin. Further research is required to test the sensibility of embryos of secondary hole nesters to dim light and its long-term effect on their condition. Finally, long-term investigations embracing several female generations breeding in conditions of natural cavity illumination are necessary to test whether there exists any interaction between cavity illumination level, nest choice, and intensity of eggshell pigmentation.

## Methods

### Study area and experimental design

The experiment was performed in 2018 in Wielkopolski National Park, near Poznań in western Poland (52°16′07″N, 16°47′53″E). The study area (48.35 ha) was covered by deciduous and mixed forests^47^. A total number of 159 nest boxes (width 12.0 cm, length 16.0 cm, height 40.0 cm; entrance diameter 3.3 cm) were evenly distributed over the study area in 2014. The side walls of each nest box were equipped with a semi-transparent, round resin window (5.0 cm) with a moveable opaque black plastic shutter. By using shutters, we could make the boxes bright (with open shutters) or dark (with closed shutters). For more detailed information on the nest box construction, see^45^. In total, we analysed data from 14 dark and 18 bright nest boxes. From the beginning of March, nest boxes were regularly monitored every 3–5 days. Trail cameras were placed in the nest boxes to determine the date of the first laid egg and the clutch size^45^.

### Photographs

**Figure 2:**
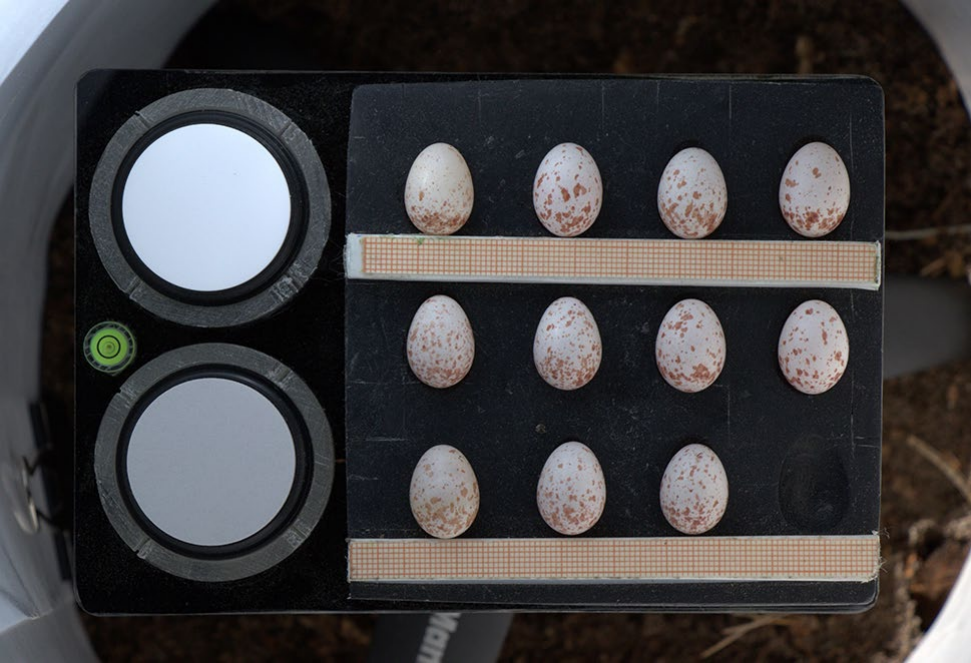
Example photo of eggs from one Great Tit clutch.

Approximately in the middle of the incubation period photographs of clutches were taken. Eggs were gently removed from the nest and placed on a black holder made of a black plastic mass and featured with a scale (mm), and reflection standards (99% and 60%, Labsphere, NH USA, Fig. 2). We took photographs only during sunny weather, in a patch of sunlight. In order to disperse light and illuminate the eggs evenly, they were surrounded with a sheet of white polytetrafluoroethylene (PTFE), 0.5 mm thick. The holder was attached to a 15-cm tripod to keep the eggs in a fixed position. If a clutch was larger than 12 eggs, two shots were taken. The photos were taken with Canon 7D Mark II SLR with Canon Zoom EF-S 18–135 mm (1.35–5.6) lens. The camera was placed on a tripod with the lens pointing down at a 90-degree angle. All shots were done with a focal length of 85 mm, aperture f/8 and ISO 640. To eliminate camera shake, we took photos with the self-timer function. For each photo, the automatic exposure bracketing function (AEB, −1, 0, +1 EV) was used and all images were saved as RAW files. After this procedure, the eggs were returned to the nest box without delay. All methods were approved by the Regional Directorate for Environmental Protection, the Ministry of Climate and Environment, and performed in accordance with Polish law.

### Digital image analysis

The collected RAW photos were screened in RAWTherapee software to select the shots with the best exposure. Then, we processed the photos in ImageJ software^48^ with MICA Toolbox plug-in, version 1.22^21^. First, we selected grey standards to normalise the images and set a scale. Next, we selected regions of interest (i.e. eggs) and recorded information about egg dimensions (length, width, volume, and surface area). Furthermore, we estimated the percentage of egg surface occupied by spots by creating a binary mask. To prepare the mask, we used a built-in ImageJ thresholding function with the following settings: 20-px radius and Phansalkar method. We worked out the settings empirically, visually comparing the mask with the original image of an egg in the green channel. We selected the green channel as it had been recognised to match the spectral sensitivity of avian double cones^49^. The average spot size was calculated using an ImageJ built-in function "Analyse Particles", and the spot reflectance was estimated by customising Batch Multispectral Image Analysis function from MICA Toolbox. This way, we computed the spot brightness as a sum of the reflectance in the red, green, and blue channels (it is thus the overall brightness in the whole range of wavelengths to which the camera is sensitive), and the red chroma of spots as the reflectance in the red channel divided by brightness. For the purpose of the analysis, spot brightness was divided by 300 to obtain values within the 0–1 range. All our custom-written macro files used during the digital image analysis are available at the GitHub repository.

### Statistical analysis

The following eggshell pigmentation characteristics were used as response variables: the percentage of spotting coverage (*percent spots*), the average size of spots (*average spot size*), the red chroma of spots (*spot Rchroma*), and the brightness of spots (*spot brightness*). Our choice was based on the results of previous studies, stating that these variables correlate significantly with protoporphyrin content^16,38^. We tested the effect of nest box type on pigmentation, while controlling for the date of the first egg laid (expressed as day number since the beginning of the year), clutch size, and eggshell thickness. The latter was calculated using the formula (1)^50,51^.

1 $$thickness = 0.005126*\left( {\frac{volume}{{1000}}*1.08} \right)^{0.456}$$

Generalised Additive Mixed Models (GAMMs)^52^ were used to test whether the nest box type affects eggshell pigmentation traits. We assumed that *percent spots*, *spot brightness,* and *spot Rchroma* originate from a beta distribution with the logit link function, while *average spot size* followed a Gaussian distribution with the logarithmic link function. The ID of the nest box was used as a random effect. The date of the first egg laid and the eggshell thickness were fitted as smooth terms, but when they were significant at 1.0 effective degrees of freedom, they were refitted as parametric terms. GAMMs were fitted using a function from the *mgcv* package^52^, diagnosed with tools from the *DHARMa* package^53^, and visualised by the plotting function provided by the *visreg* package^54^. Within-clutch repeatability of egg dimensions, *average spot size*, as well as arcsine-transformed *percent spots*, *spot brightness,* and *spot Rchroma* was calculated as an intra-class correlation coefficient, using the LMM-based approach for Gaussian data and functions from the *rptR* package^55^. Statistical analyses were conducted in R 4.2 software^56^.

## Supplementary Information

Supplementary Information.

## Data Availability

Data are deposited in the Zenodo archive https://doi.org/10.5281/zenodo.7653168. All our custom-written macro files used during the digital image analysis are available at the GitHub repository https://github.com/KlaudiaSzala/eggshell-spots.
