# Supplementary material for: Effect of light intensity in the nest site on eggshell pigmentation in a hole-nesting passerine
Source: Sci Rep. 2023 Jun 16;13:9764. doi: 10.1038/s41598-023-36658-4 (PMC10276044; doi:10.1038/s41598-023-36658-4)
Supplement: Supplementary file 1 — Supplementary Information. [file 41598_2023_36658_MOESM1_ESM.pdf]

SUPPLEMENTARY INFORMATION

EFFECT OF LIGHT INTENSITY IN THE NEST SITE ON EGGSHELL PIGMENTATION IN A HOLE-NESTING PASSERINE

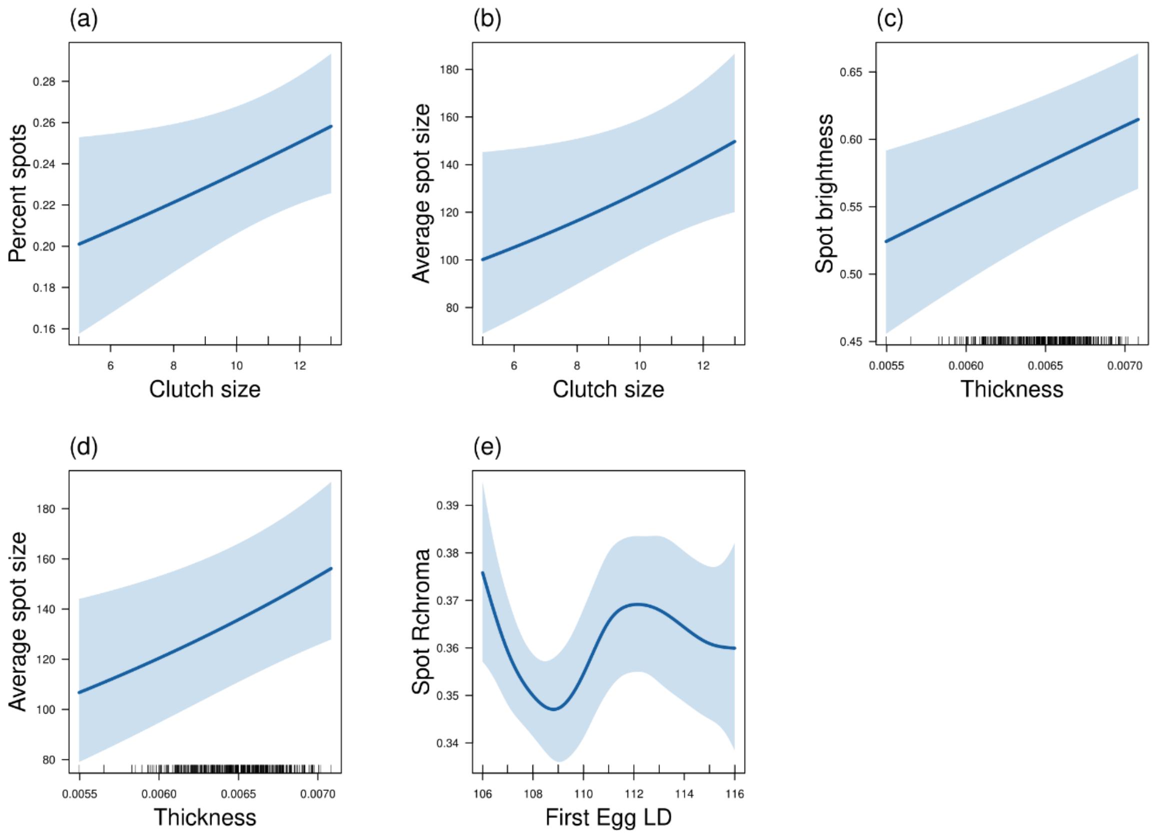

Supplementary Figure S1 (a)-(e). Conditional plots visualising significant effects of Great Tit egg characteristics on pigmentation traits presented on a response scale. Shaded areas indicate 95% confidence intervals, while tick marks represent data points. LD = laying date.
